# Prevalence and factors associated with adverse drug events among patients on dolutegravir-based regimen at the Immune Suppression Syndrome Clinic of Mbarara Regional Referral Hospital, Uganda: a mixed design study

**DOI:** 10.1186/s12981-022-00442-7

**Published:** 2022-04-02

**Authors:** Angella Namulindwa, John Hans Wasswa, Winnie Muyindike, Robert Tamukong, Joseph Oloro

**Affiliations:** 1 Department of Pharmacy, Faculty of Medicine, Mbarara University of Science and Technology, P.O. Box 1410, Mbarara, Uganda; 2 School of Public Health, Clarke International University, P.O.Box 7782, Kampala, Uganda; 3 Immune Suppression Syndrome Clinic, Mbarara Regional Referral Hospital, P.O. Box 40, Mbarara, Uganda; 4 Department of Pharmacology and Therapeutics, Faculty of Medicine, Mbarara University of Science and Technology, P.O. Box 1410, Mbarara, Uganda

**Keywords:** Dolutegravir-based, Adverse drug events, HIV, Uganda

## Abstract

**Background:**

In low income countries such as Uganda progress has been made towards achieving the United Nations AIDS programme 95-95-95 target however efforts are still impeded by pretreatment drug resistance and adverse drug events (ADEs) hence introduction of dolutegravir-based antiretroviral therapy as first-line treatment due to a higher genetic barrier to resistance, better tolerability and safety profile. However, recent studies have raised concerns regarding its safety in real-clinical settings due to ADEs and being a recently introduced drug there is need to actively monitor for ADEs, hence this study aimed to establish the prevalence and factors associated with ADEs among patients on dolutegravir-based regimen at the Immune Suppression Syndrome (ISS) Clinic- Mbarara Regional Referral Hospital (MRRH).

**Methods:**

A mixed design study was conducted at ISS Clinic-MRRH among 375 randomly selected patients who had been exposed to DTG-based regimen for at-least 12 weeks. These were interviewed to obtain data on socio-demographics, dietary habits and their files reviewed for ADEs. Data entry was done using Epi-data 3.0 and exported to SPSS 25.0 for analysis. Prevalence was determined as a percentage, and ADE associated factors assessed using bivariate analysis, those found significant were further subjected to multivariate analysis and considered significant at P < 0.05.

**Results:**

The prevalence of ADEs among patients on DTG-based regimen was found to be 33.1% (124/375) with 5.6% (7/124) participants discontinued from treatment due ADEs, 4 due to hyperglycemia and 3 liver toxicity. The commonly experienced ADE was allergy at 36.3%. Male sex (AOR 1.571, 95% CI 1.433–1.984), WHO stage one at entry to care (AOR 4.586, 95% CI 1.649–12.754), stage two (AOR 4.536, 95% CI 1.611–12.776), stage three (AOR 3.638, 95% CI 1.262–10.488), were significantly associated with ADEs. Patients with undetectable viral load at initiation of DTG-based regimen were 67.6% less likely to experience ADEs (AOR = 0.324, 95% CI 0.1167–0.629).

**Conclusion:**

This study reports a prevalence of 33.1% of ADEs among patients on DTG-based regimen. The most commonly experienced ADE was allergy. Male sex, early HIV disease stage at entry into care and detectable viral load at initiation of DTG-based regimen were significantly associated with ADEs. It is crucial to actively monitor patients with these characteristics for ADEs.

## Background

The Joint United Nations Programme on HIV/AIDS (UNAIDS) in 2014 launched the 95-95-95 strategy aimed to end the AIDS epidemic by 2030 through achieving 95% diagnosed of all people living with HIV (PLWHIV), 95% on antiretroviral therapy (ART) among diagnosed and 95% virally suppressed among treated [1]. Globally by 2020, 84% of PLWHIV knew their status, 87% were accessing ART and of these 90% were virally suppressed [2]. In Uganda 89% were aware of their status, 84% of PLWHIV were enrolled on ART and 90% were virally suppressed [3].

Progress towards achieving the 95-95-95 target has been made however, a rise in pretreatment drug resistance to first-line ART and adverse drug events (ADEs) among others pose a serious threat to strides made in low income countries such as Uganda hence need for more potent ART with higher genetic barrier to drug resistance, better safety and toxicity profile [4, 5].

Following World Health Organization (WHO) recommendations in 2018, dolutegravir (DTG)-based ART was adopted as first-line treatment for all PLWHIV in Uganda [6, 7]. Dolutegravir, an integrase inhibitor with a higher genetic barrier to resistance, excellent tolerability, low potential for drug interaction and more effectiveness, is combined with two nucleoside/tide reverse transcriptase inhibitors usually abacavir/lamivudine or tenofovir/lamivudine [6, 8].

In low income countries such as Uganda where dolutegravir-based ART has recently been introduced there is need for active monitoring for potentially new and emerging adverse drug events [9]. Clinical trials conducted on DTG-based regimen reported frequency of occurrence of ADEs at 2%, however recent studies in real clinical settings have reported a much higher frequency of up to 10% [10, 11]. A study conducted in central Uganda to assess acceptability and viral suppression of DTG-based first-line ART reported that 32% patients experienced ADEs [12]. Patients on DTG-based regimen may experience ADEs including nausea, vomiting, diarrhea, allergies, rash, headache, insomnia, hepatotoxicity and hyperglycemia among others [10, 13–15]. Studies have reported various factors associated with adverse drug events among patients on DTG-based regimen, among which include female sex and age at entry into study [8, 14].

There is scarcity of information on ADEs of DTG-based regimen in countries where it has recently been introduced such as Uganda, therefore, this study aimed to determine the prevalence and factors associated with adverse drug events among patients on dolutegravir-based regimen at the Immune Suppression Syndrome (ISS) Clinic- Mbarara Regional Referral Hospital (MRRH).

## Methods

### Study design

This research was a mixed design study involving patients in HIV care at the Immune Suppression Syndrome Clinic of Mbarara Regional Referral Hospital, Uganda who had been exposed to dolutegravir-based antiretroviral therapy for at least 12 weeks. The objectives of the study included, to determine the prevalence of adverse drug events, identify adverse drug events experienced and establish factors associated with adverse drug events among patients on dolutegravir-based regimen at the ISS Clinic-MRRH.

The ethical considerations of this study were approved by Mbarara University of Science and Technology Research Ethics Committee (MUST-REC) approval number, MUREC 1/9 02/12-19, and Faculty of Medicine through the Faculty Research Committee (FRC) approval number DMS 6.

### Study setting

This study was conducted at the Immune Suppression Syndrome Clinic of the Mbarara Regional Referral Hospital, a government-aided hospital located in Mbarara district in the South western region of Uganda. The facility majorly serves patients from South-Western districts of Uganda including Buhweju, Bushenyi, Ibanda, Isingiro, Kazo, Kiruhura, Mitooma, Ntugamo, Mbarara, Rubirizi, and Rwampara among others. Currently, the ISS clinic serves a total number of 21,600 patients; 11,600 pediatric and 10,000 adults. The facility has an average daily attendance of 300 patients. The ISS clinic provides services including; HIV counselling and testing, elimination of mother to child transmission of HIV, HIV care, treatment and support for people living with HIV/AIDs including children, adolescents and adults.

### Study population and sample

The study population consisted of both male and female patients aged 20 years and above at the time of initiation of dolutegravir-based HAART regimen and had been on the regimen for at least 12 weeks at the Immune Suppression Syndrome Clinic of Mbarara Regional Referral Hospital, Uganda.

The sample size of 375 patients was determined using the Slovin’s formula [16] based on the total population of patients who were on dolutegravir-based ART regimen at ISS clinic.

Unique identification codes for eligible participants were run through Microsoft excel and a sample size of 375 was generated by random draw method. An appointment list was used to identify when the patients attended clinic visit, upon which they were asked to consent and participated in the study. The method eliminated bias, provided an equal chance to every eligible patient to be selected for the study and patients participated on their official clinic appointment day.

Written and informed consent was obtained from the patients to participate in the study and to use their files for obtaining data for the study. Before participants signed consent forms, they were informed that participation was voluntary and they could drop out at any time, the purpose, objectives, possible benefits and risks of the study were clearly explained and only patient identification numbers were used which maintained utmost confidentiality.

### Data collection tool and procedures

Selected patients were interviewed, there-after their medical files reviewed and data on ADEs was obtained from the ART cards as recorded by clinicians. The data collection tool consisted of two sections; The first section was completed through patient interview and collected information on social demographics including; sex, age, marital status, religious affiliation, level of education and employment status, types of meals consumed before swallowing medicine, if patients received counselling instructions to follow while taking the regimen and time the medicine is taken.

The second section was completed by file review and collected data on duration since HIV diagnosis, CD4 at entry into care, duration on HAART, viral load at initiation of dolutegravir-based regimen, previous ART regimen, body mass index, recorded adverse drug event since start of dolutegravir-based regimen, any treatment modification; discontinuation, comorbidities, other medications, blood glucose measurements and liver function tests.

The data abstraction form for each patient/file was assigned an identification code. The filled: forms were checked for accuracy, consistency and completeness by the principal investigator. Completed forms were kept under restricted access which protected patient confidentiality and data from alteration. The first section of the data collection tool was translated to Runyankore the commonly used local language.

### Statistical analysis

All filled data collection forms were checked, coded and data entry done using Epi data 3.0. Data cleaning and validation was done to detect any errors. The data was exported and analyzed using statistical package for social sciences (SPSS) version 25.0. Socio-demographics were presented using descriptive statistics and categorical variables were presented using frequencies and percentages. The data was presented using text, tables, and graphs. Prevalence of ADEs was determined by obtaining the number of patients in the sample who had at least one ADE recorded in their medical file on the ART card divided by the total number of the sample size, expressed as a percentage.

Adverse drug events experienced by patients were captured from ART cards in the medical files as recorded by clinicians. Severity rating of ADEs was based on the DAIDS grading of ADEs using data from medical files [17]. ADEs were graded 1 (mild) if symptoms caused no or minimal interference with usual social activities with intervention not indicated, 2 (moderate) if symptoms caused greater than minimal interference with usual social and functional activities with interventions indicated, 3 (severe) if symptoms caused inability to perform usual social and functional activities with intervention or hospitalization indicated and 4 (potentially life threatening) if symptoms caused inability to perform basic self-care functions with intervention indicated to prevent permanent impairment, persistent disability, or death. This data was presented in form of a graph and tables.

The relationship between factors associated with adverse drug events to dolutegravir-based regimen was established using bivariate analysis. Variables found significant at bivariate level were then subjected to multivariate analysis. Variables were considered statistically significant if p-value was less than 0.05 measured with odds ratio at 95% confidence interval.

## Results

### Characteristics of study participants

Most of the study participants were male 59.5% (223/375), with majority of respondents in the age bracket of 40–49 years and 50–59 years consisting of 34.1% (128/375) each, with median age 49 years interquartile range 12. Participants who had been on HAART for 5–10 years were 78.9% (296/375), 77.9% (292/375) of the participants had undetectable viral loads at the time of initiation of DTG-based regimen, and all the participants were in WHO stage one at initiation of DTG-based regimen (Table 1).

**Table 1:** Social demographic characteristics of study participants on dolutegravir-based regimen at the ISS clinic- MRRH

Variables	All patients N = 375
Sex (n [%])	
Male	223 [59.5]
Female	152 [40.5]
Age (n [%])	
20–29 years	16 [4.3]
30–39 years	55 [14.7]
40–49 years	128 [34.1]
50–59 years	128 [34.1]
≥ 60 years	48 [12.8]
Marital status (n [%])	
Single	57 [15.2]
Married	219 [58.4]
Separated/divorced	36 [9.6]
Widow/widower	63 [16.8]
Highest education level (n [%])	
No formal education	43 [11.5]
Primary	184 [49.1]
‘O’level	91 [24.2]
‘A’level	18 [4.8]
Tertiary	39 [10.4]
Employment status (n [%])	
Employed	282 [75.2]
Unemployed	93 [24.8]

n, number of participants; %, percentage; ≥ , greater than or equal to; ‘O’level, ordinary level; ‘A’level, advanced level, ISS, Immune suppression syndrome; MRRH, Mbarara regional referral hospital

### Prevalence of ADEs

One third (33.1%, 124/375) of the respondents had at least one ADE recorded in their files since initiation of DTG-based regimen.

### ADEs experienced by study participants

**Fig. 1:**
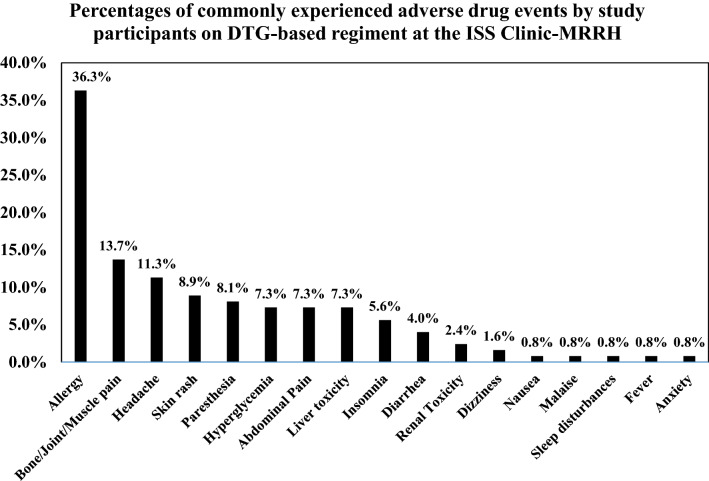
Commonly experienced ADEs by study participants on dolutegravir-based regimen at the ISS clinic-MRRH. ADE, adverse drug event; %, percentage (number of participants with ADE recorded/ total number of participants with at least one ADE recorded in their medical files)

The commonly recorded ADEs included; abdominal pain, hyperglycemia and hepatotoxicity each at 7.3%, paresthesia at 8.1%, headache at 11.3% allergy at 36.3% (Fig. 1).

Using the DAIDs grading of adverse drug events, of the 7.3% (9/124) patients who experienced hyperglycemia, five had grade 1, two had grade 2, one with grade 3 and one with grade 4, this was reported between 13 and 62 weeks of starting the DTG-based regimen.

Of the 7.3% (9/124) patients who experienced liver toxicity; eight had grade one and one had grade 4, this was reported between 15 and 63 weeks of starting the DTG-based regimen. All the patients who experienced liver toxicity were concomitantly taking isoniazid preventive therapy.

As result of ADEs 5.6% (7/124) participants were discontinued from DTG-based regimen, 4 due to hyperglycemia and 3 liver toxicity.

### Factors associated with ADEs

At bivariate analysis male sex (Crude OR = 1.789, 95% CI 1.156–2.768), being in the age bracket of 30–39 years (Crude OR = 2.621, 95% CI 1.089–6.307), being married (Crude OR = 2.627, 95% CI 1.475–4.679) and being employed (Crude OR = 1.674, 95% CI 1.032–2.716), eating non-fatty meals before swallowing the medicines (Crude OR = 0.571, 95% CI 0.335–0.976), duration of HIV diagnosis of less than 5 years (Crude OR = 1.789, 95% CI 1.156–2.768), having HIV for 5–10 years since diagnosis (Crude OR = 3.417, 95% CI 1.327–8.795), being in WHO stage one at entry into care (Crude OR = 4.472, 95% CI 1.757–11.386), being in WHO stage two at entry into care (Crude OR = 4.000, 95% CI 1.539–10.396), being in WHO stage three at entry into care (Crude OR = 2.800, 95% CI 1.050–7.469), having undetectable viral load at initiation of DTG (Crude OR = 0.336, 95% CI 0.18–0.625), were significantly associated with ADE among patients on DTG-based regimen (Table 2).

**Table 2:** Bivariate and multivariate analysis of factors associated with adverse drug events among study participants on dolutegravir-based regimen at ISS clinic-MRRH

Variables	Recorded experience of ADE		Bivariate odds ratio (95% CI)	p-value	Multivariate odds (95% CI)	p-value
	Yes (%)	No (%)				
Sex						
Male	62 (50.0)	161 (64.1)	1.789 (1.156–2.768)	0.009*	1.571 (1.433–1.984)	0.031*
Female	62 (50.0)	90 (35.9)	1.0		1.0	
Age						
20–29 years	5 (4.0)	11 (4.4)	1.441 (0.432–4.810)	0.552		
30–39 years	11 (8.9)	44 (17.5)	2.621 (1.089–6.307)	0.032*		
40–49 years	44 (35.5)	84 (33.5)	1.251 (0.631–2.478)	0.521		
50–59 years	45 (36.3)	83 (33.1)	1.208 (0.610–2.392)	0.587		
≥ 60 years	19 (15.3)	29 (11.6)	1.0			
Employment status						
Employed	85 (68.5)	197 (78.5)	1.674 (1.032–2.716)	**0.**037*		
Unemployed	39 (31.5)	54 (21.5)	1.0			
Meals eaten before swallowing medicines						
Non-fatty meals	35 (28.2)	50 (19.9)	0.571 (0.335–0.976)	0.040*		
Plant based	11 (8.9)	22 (8.8)	0.800 (0.363–1.762)	0.580		
Animal based	24 (19.4)	44 (17.5)	0.733 (0.407–1.322)	0.302		
Plant and animal	54 (43.5)	135 (53.8)	1.0			
Duration since HIV diagnosis						
< 5 years	12 (9.7)	41 (16.3)	3.417 (1.327–8.795)	0.011*		
5–10 years	27 (21.8)	93 (37.1)	3.444 (1.525–7.779)	0.003*		
11–15 years	69 (55.6)	101 (40.2)	1.464 (0.686–3.122)	0.324		
> 15 years	16 (12.9)	16(6.4)	1.0			
WHO staging at entry into care						
Stage one	45 (36.3)	115 (45.8)	4.472 (1.757–11.386)	0.002*	4.586 (1.649–12.754)	0.004*
Stage two	35 (28.2)	80 (31.9)	4.000 (1.539–10.396)	0.004*	4.536 (1.611–12.776)	0.004*
Stage three	30 (24.2)	48 (19.1)	2.800 (1.050–7.469)	0.040*	3.638 (1.262–10.488)	0.017*
Stage four	14 (11.3)	8 (3.2)	1.0		1.0	
Viral load at initiation of DTG-based regimen						
Undetectable	110 (88.7)	182 (72.5)	0.336 (0.180–0.625)	0.001*	0.324 (0.1167–0.629)	0.001*
Detectable	14 (11.3)	69 (27.5)	1.0		1.0	

Statistically significant P values less than 0.05 of ADE associated factors at multivariate analysis including sex, WHO staging at entry into care and viral load at initiation of DTG-based regimen

^*^Significance, less than 0.05; %, percentage; ADE, adverse drug event; COR, crude odds ratio; AOR, Adjusted odds ratio; CI, confidence interval; HIV, human immunodeficiency virus; WHO, world health organization; DTG, dolutegravir; ART; < , less than; > , greater than; ≥ greater than or equal to

At multivariate analysis, all variables found significant at bivariate analysis were considered. Male sex (Adjusted OR = 1.571, 95% CI 1.433- 1.984), being in WHO stage one at entry to care had (AOR = 4.586, 95% CI 1.649–12.754) WHO stage two (AOR = 4.536, 95% CI 1.611–12.776), WHO stage three (AOR = 3.638, 95% CI 1.262–10.488), and viral load at initiation of DTG-based regimen were significantly associated with ADEs among patients on DTG-based regimen. Patients with undetectable viral load at DTG-regimen initiation (AOR = 0.324, 95% CI 0.1167–0.629) were less likely to experience ADEs (Table 2).

## Discussion

There is limited information on adverse drug events of dolutegravir-based regimen, more so in low income countries such as Uganda where DTG-based regimens have been recently introduced and hence a need to actively monitor for any emerging ADEs [9]. In this study we found that up to a third of all participants had at least one ADE recorded which is comparable to findings in a study conducted in Central Uganda [12]. The findings in this study are higher than those in Northeastern Brazil [11]. The difference might be because the study in Brazil was conducted among patients whose first ART regimen contained DTG whereas in this study majority of participants had already been on other ART regimen. Our study findings further strengthen the need for active monitoring of ADEs among patients on DTG-based regimens.

We observed that allergy was the most commonly recorded ADE much higher than reports from studies in Switzerland and France [8, 18], possibly because of variation in study setting and populations characteristics. It is important to note that in this study there were no exhaustive medical notes on ADEs hence proper differentiation of allergy types could not be made, possibly contributing to the high number reported. This study reports headache as the most common neuropsychiatric ADE comparable to reports from a study in Netherland [19]. However, much higher than findings in German and Brazil respectively [10, 11] possibly due to variation in study populations and settings. It is suggested that there is free passage of DTG across the blood–brain barrier however it is still unknown whether lower central nervous system drug exposure would mitigate potential neurotoxicity [20]. We observed that 7.3% patients developed hyperglycemia contrary to findings of 0.47% reported in another study conducted in Uganda [13]. This difference might be because in the later study a larger study population was followed up over a period of 12 months whereas in the former study results of a smaller study population were obtained from already made records from patient files. DTG interferences with cellular level insulin signaling and causes defects in lipid metabolism that result in obesity which may lead patients into developing insulin resistance and ultimately increased blood glucose levels [21]. In this study liver toxicity occurred in 7.3% patients, all of whom were concomitantly taking isoniazid at time of experiencing ADE which is comparable to findings from a study conducted in Switzerland [8]. Co-administration of DTG-based therapy and isoniazid results in significantly elevated levels of inflammatory markers such as c-reactive protein, interferon-γ, CXCL10, and other cytokines which result into liver toxicity [22].

Findings from our study reveal that male sex was significantly associated with ADEs which is contrary to findings from studies in Switzerland and Spain who found female sex significantly associated with ADEs [8, 23]. This is possibly due to differences in genetic and physiological variations in study populations. In this study participants with an early WHO disease stage at entry into care; I, II and III were 4.586, 4.536 and 3.638 times respectively more likely to experience ADEs than those in stage IV however this is contrary to a study conducted in Ethiopia which reported that ADEs were 7 times more likely to occur among patients in WHO disease stage IV than in I [24]. This is probably because of differences in ART regimen administered in the two studies and variation in study population characteristics. Lastly, patients who had undetectable viral load at initiation of DTG-based regimen were 67.6% less likely to have ADEs compared to those with a detectable viral load probably because the former have a better immunity hence are less susceptible to experiencing ADEs whereas the later have compromised immunity hence more susceptible to ADEs. These findings are consistent with those of a study in France which reported that a higher viral load at start of the ART regimen was associated with occurrence of ADEs [25].

## Conclusion

This study reports a prevalence of 33.1% of ADEs among patients on DTG-based regimen. The most commonly experienced ADE was allergy. Male sex, early WHO HIV disease stage at entry into care and a detectable viral load at initiation of DTG-based regimen were significantly associated with ADEs. It is crucial to actively monitor patients with these characteristics for ADEs. Therefore, we recommend regular screening and thorough investigation of adverse drug events among all patients on dolutegravir-based therapy especially among male patients with a detectable viral load at ignition of DTG-based regimen as well as conducting of blood glucose and liver function tests at initiation of DTG-based regimen and periodically especially in the third month (13 weeks) of starting therapy and 16 months (63 weeks) of DTG-based therapy.

## Study limitations

The data on adverse drug events such as allergies was captured from medical files as recorded by clinicians hence thorough and proper investigation on each ADE could not be conducted.

Existing laboratory results on blood glucose tests and liver function tests were used to capture laboratory data on adverse drug events such as hyperglycemia and liver toxicity however most patient files did not have these test results.

## Data Availability

All data generated or analyzed during this study are included in this published article.

## Abbreviations

ADEs: Adverse Drug Events
AIDS: Acquired Immunodeficiency Syndrome
ALT: Alanine aminotransferase
AOR: Adjusted Odds Ratio
ART: Antiretroviral therapy
ARVs: Antiretrovirals
AST: Aspartate aminotransferase
DAIDS: Division of Acquired Immunodeficiency Syndrome
DTG: Dolutegravir
FRC: Faculty Research Committee
HAART: Highly Active Anti-Retroviral Treatment
ISS: Immune Suppression Syndrome
IRB: Institutional review board
MOH: Ministry of Health
MRRH: Mbarara Regional Referral Hospital
MUST: Mbarara University of Science and Technology
PLWHIV: People Living With HIV
REC: Research Ethics Committee
SPSS: Statistical package for social sciences
UNAIDS: United Nations Programme on HIV/AIDS
WHO: World Health Organization
